# Environmental judicature and firm productivity: Evidence from a quasi-natural experiment

**DOI:** 10.1371/journal.pone.0317037

**Published:** 2025-01-24

**Authors:** Xiaoqi Huang, Wenbo Yao, Zhi Cao

**Affiliations:** 1 School of Economics, Zhongnan University of Economics and Law, Wuhan City, Hubei Province, China; 2 School of Accounting, Zhongnan University of Economics and Law, Wuhan City, Hubei Province, China; University of International Business and Economics, CHINA

## Abstract

The impact of environmental governance on firm productivity has been widely discussed, but few studies have examined the function of environmental judicature. Using the establishment of environmental courts as a quasi-natural experiment, this paper examines the relationship between environmental judicature and firm productivity. Our findings show that environmental courts will reduce firm productivity in the short term, chiefly owing to the increased environmental violation costs, environmental reputation costs and environmental compliance costs. The conclusion remains robust after mitigation of heterogeneous treatment effects, PSM-DID estimation, placebo tests and IV estimation. This negative effect is more pronounced for firms located in regions with high legal scores and low government intervention, for firms located in the eastern region, for firms with weak market power, and for firms with high pollution intensity. In addition, our further analysis suggests that environmental courts would improve the long-term firm productivity and regional green productivity, indicating that strengthening environmental judicature are conducive to firms’ sustainable growth and regions’ green transformation in the long run.

## 1. Introduction

As environmental issues worsen, the pursuit of a harmonious equilibrium between environmental governance and economic performance has emerged as a prominent research focal point. China, as one of the world’s largest economies, has undergone significant economic advancement over the past few decades. However, such rapid development has caused environmental degradation and pollution [1–3]. The traditional model of extensive economic development, which heavily relies on resource consumption and pollution emissions, is not sustainable. The pursuit of high-quality development which seeks to attain a balance between economic growth and environmental protection has become an important discussion topic.

Firms are key drivers of economic growth as well as primary polluters, yet many are hesitant to invest in environmental protection owning to the perceived cost burden and potential loss of profits. As a result, enhancing firms’ environmental governance and promoting green transformation has become crucial in achieving high-quality development. China has formulated a well-developed legal system for environmental protection, but it has limited effectiveness. Since economic growth is an important criterion for promotion, local Chinese governments may safeguard heavily polluting firms and impede the enforcement of environmental governance [4, 5], as these firms make substantial contributions to employment, tax revenue and local GDP. Hence, environmental governance requires a third-party supervision, particularly environmental judicature. The establishment of environmental courts is an initiative aiming at enhancing supervision regarding environmental judicature, which are designed to adjudicate environmental cases, expedite legal proceedings, and improve environmental litigation enforcement. Environmental courts have been demonstrated to increase firms’ environmental investment and spur green innovation, while also reduce regional pollution emissions and improve environmental quality [6–8]. However, it remains unclear if environmental courts will reduce firm productivity due to additional cost burden? Could a win-win situation between environmental protection and economic development be achieved? Answers to the above questions are of critical significance for policymakers and entrepreneurs aiming to attain both economic growth and environmental sustainability.

In this paper, based on a quasi-natural experiment of the establishment of environmental courts in China, we try to explore the effect of environmental judicature on firm productivity. The first environmental court was established in QingZhen in 2007, which aimed at dealing with local water pollution issue. Since then, the establishment of environmental courts has boomed. By 2014, 43 intermediate people’s courts have been established. Considering that it evidently intensifies the supervision of firms’ environmental violations, we anticipate that firms will reshape production planning, investment strategy, and resource allocation to respond to potential risks of environmental litigation. Specifically, on the one hand, firms will allocate more resources to environmental sustainability, which results in an increase in firms’ cost burden, thus reducing firm productivity in the short term. On the other hand, external judicial supervision can reduce the short-sightedness of managers, induce firms to strengthen environmental governance through green innovations and transformations, which may improve firm productivity in the long term.

Our research finds that: (1) Environmental courts will reduce firm productivity in the short term owning to the increased environmental violation costs, environmental reputation costs and environmental compliance costs, which remains solid after mitigation of heterogeneous treatment effects, PSM-DID estimation, placebo tests, IV estimation and a series of robust checks. (2) This negative effect is more pronounced for firms located in regions with high legal scores and low government intervention, for firms located in the eastern region, for firms with weak market power, and for firms with high pollution intensity. (3) In addition, our further analysis finds that the environmental courts would improve the long-term firm productivity and regional green productivity, indicating that the environmental courts are conducive to firms’ sustainable growth and regions’ green transformation in the long run.

Compared with existing research, we made some marginal contributions as follows: first, we investigate the effect of environmental governance on firm productivity from the perspective of environmental judicature. Earlier research primarily examines environmental litigation and environmental administration enforcement [9–12], and few of them examine the topic from the perspective of environmental judicature. We exploit the establishment of environmental courts in China as a quasi-natural experiment to examine the relationship between environmental judicature and firm productivity, thereby contributing to clarify how will environmental judicature affect firm development. Furthermore, in order to address endogeneity concerns comprehensively, we employ the ventilation coefficient and thermal inversion as instrumental variables, mitigating the bias of heterogeneous treatment effects, alongside conducting PSM-DID estimation, placebo tests, and a battery of robustness checks. These measures are conducive to obtaining unbiased estimates of the relationship between environmental judicature and firm performance.

Second, we examine the inter-temporal effect of environmental judicature on firm productivity. Various literature has examined how environmental governance affect firm productivity, but the outcomes of these studies are inconsistent. Some argue that environmental governance leads to a decrease in firm productivity due to environmental compliance costs and the crowding-out of firms’ productive investment [13–15]. However, other studies argue that firms can enhance resource utilization efficiency and promote innovation under environmental regulation pressure [16, 17], which could offset the environmental compliance costs [18, 19] and spur firm productivity growth [20, 21]. Drawing from the prior discussions, this paper tries to reconcile the conflicting theories by analyzing the inter-temporal heterogeneity effect between environmental judicature and firm productivity, since firms’ green transformation is a prolonged process that cannot be achieved overnight.

Third, our paper may contribute to the study on the impact of environmental supervisory agency, such as environmental courts. Prior research has demonstrated that environmental courts increase firm environmental investment and encourage green innovation, while also leading to reductions in pollution emissions [6–8]. These findings suggest that environmental courts serve as a powerful means of promoting environmental governance. However, the effect of environmental courts on firm productivity and their potential role in impeding economic development remain unclear. Our study aims to shed light on these issues by examining how environmental courts affect firm productivity.

The following parts of the paper are organized as follows. The institutional background and theoretical analysis section outlines the establishment process of environmental courts, their role, and provides a theoretical framework on how environmental judicature influences firm productivity. The data and identification strategy section introduces the data and sample, explains the empirical identification strategy, and provides a statistical description. The results section presents the baseline results, followed by an endogeneity analysis, additional robustness checks, an exploration of potential mechanisms, and a heterogeneity analysis. The further analysis section examines the inter-temporal effects of environmental judicature on firm productivity and explores how environmental judicature influences regional green productivity. The conclusions and policy implications section summarizes the study’s findings and offers policy recommendations from the perspectives of legal institutions, local governments, and firms, respectively.

## 2. Institutional background and theoretical analysis

### 2.1. Institutional background

With the rapid development of industrialization and urbanization, China confronts serious problems of environmental pollution and ecological degradation [1–3]. Since the enactment of The Environmental Protection Law of the People’s Republic of China in 1989, China has developed a well-developed environmental protection legal system to strengthen environmental governance. However, inefficiencies in environmental judicature hinders resolving numerous environmental disputes through the judicial system [22]. This is mainly because of the unique features of environmental cases, which encompass various litigation types, including civil, administrative, and criminal cases, as well as the challenges in evidence gathering and causal inference. Thus, the complexity of environmental cases leads to higher demands on the professionalism of judges; otherwise, it may result in inefficiencies within environmental judicature. The establishment of environmental courts is a potent institutional arrangement to achieve the specialization of environmental judicature and improve trial efficiency in environmental cases [23, 24].

**Fig 1:**
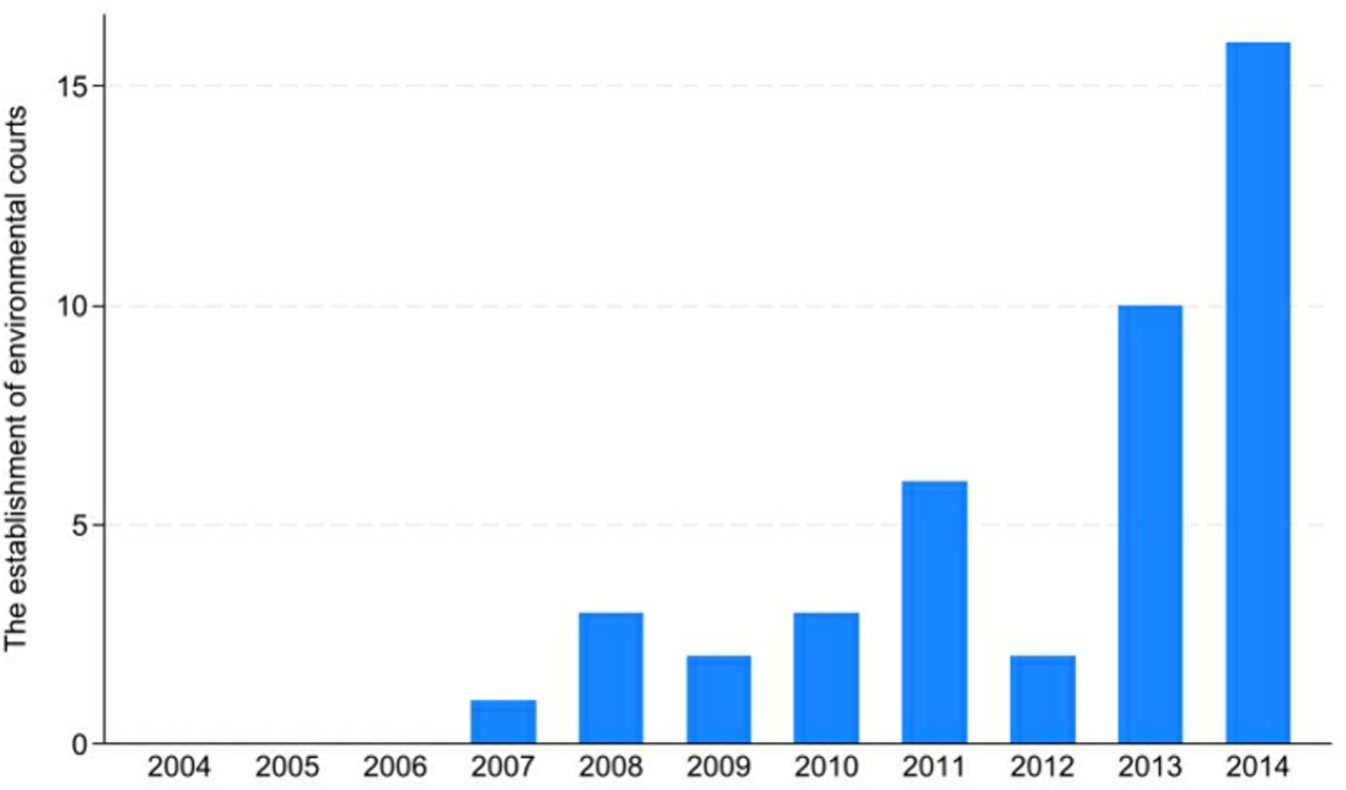
The establishment of environmental courts.

In 2007, Qingzhen established the first environmental court to address significant water pollution. Subsequently, various regions also established environmental courts [25, 26]. As of 2014, 43 environmental courts have been established at the city level. Fig 1 depicts the annual increase in the number of newly established environmental courts. We can find more environmental courts are established since 2007, indicating that environmental judicature is strengthening over the period.

There are several advantages when environmental courts handle environmental dispute cases. First, environmental courts have a team of professionals, including the well-trained judges and environmental experts, which helps in the evidence review and casual determination. Second, the trial mode of the combination of administration, civil and criminal improves the efficiency and rationality of case trial. Third, environmental courts are convenient for public interest litigation on environmental disputes, which strengthens public supervision through judicature channel [24]. Fourth, the cross-regional jurisdiction model contributes to maintaining judicial independence, which mitigates the intervention of local governments. The cross-regional jurisdiction model is a novel model for delineating jurisdictional regions based on ecosystems or ecologically functional regions (e.g., watersheds, forested regions or mountain ranges), in contrast to the traditional model based on administrative boundaries. Thus, it reduces local government’s interference in the judiciary and decreases the scope for favouritism towards highly polluting firms [6, 8, 27]. In summary, we argue that environmental courts guarantee environmental judicial efficiency and strengthen environmental judicature.

### 2.2. Theoretical analysis

The effectiveness of environmental legislation requires an efficient environmental judicature system. As an important mechanism to promote environmental judicature in China, environmental courts will safeguard environmental judicial authority and better serve environmental protection. Given that firms are the principal contributors to environmental pollution [6], it is believed that environmental courts will impose greater environmental protection pressure and environmental cost burden on firms.

In the short term, firms can only passively react and absorb the cost burden, and environmental courts may reduce firm productivity. First, environmental courts enhance the judicial supervision of firm environmental behavior, thereby, resulting in a rise in firm environmental violation costs. More environmental disputes will be appealed to environmental courts due to the higher efficiency of environmental judicature, and firms that violate environmental laws are more likely to face environmental litigation. A more professional judicial team can also more accurately determine firms’ liability for environmental violations. Thus, firms will bear more environmental litigation costs, environmental fines and environmental remediation expenditure. Higher environmental violation costs will increase the environmental protection burden on firms, decrease their profits and productivity in the short term.

Second, environmental courts significantly enhance public environmental supervision, thereby increasing firms’ environmental reputation costs. Environmental courts provide an institutional basis for the implementation of environmental public interest litigation, stimulating public participation in environmental supervision. Furthermore, once the public discovers environmental non-compliance, the spillover effect of public scrutiny can damage firms’ reputation and image, which in turn increases the reputational costs. Damage to a firm’s reputation can affect stakeholders’ perceptions of a firm’s sustainability, which can increase financing costs [28], reduce market share [29], and undermine supply chain stability [30]. Thus, environmental courts will lead to stricter public scrutiny and increase environmental reputation costs. Higher environmental reputation costs will disrupt business operations, decrease firm profits and productivity in the short term.

Third, environmental courts may increase firm environmental compliance costs, thus reducing short-term productivity. First, to mitigate the risk of environmental litigation, firms might take a more proactive approach in paying environmental protection fees, such as pollution discharge fees and afforestation fees. These additional expenses could diminish firm profits. Furthermore, there might be increased investment in environmental protection to curb pollution emissions. The resources devoted to environmental protection may crowd out productive investment and disrupt optimal production plans of firms. Thus, environmental courts can reduce firm productivity in the short term due to increased compliance costs.

**Fig 2:**
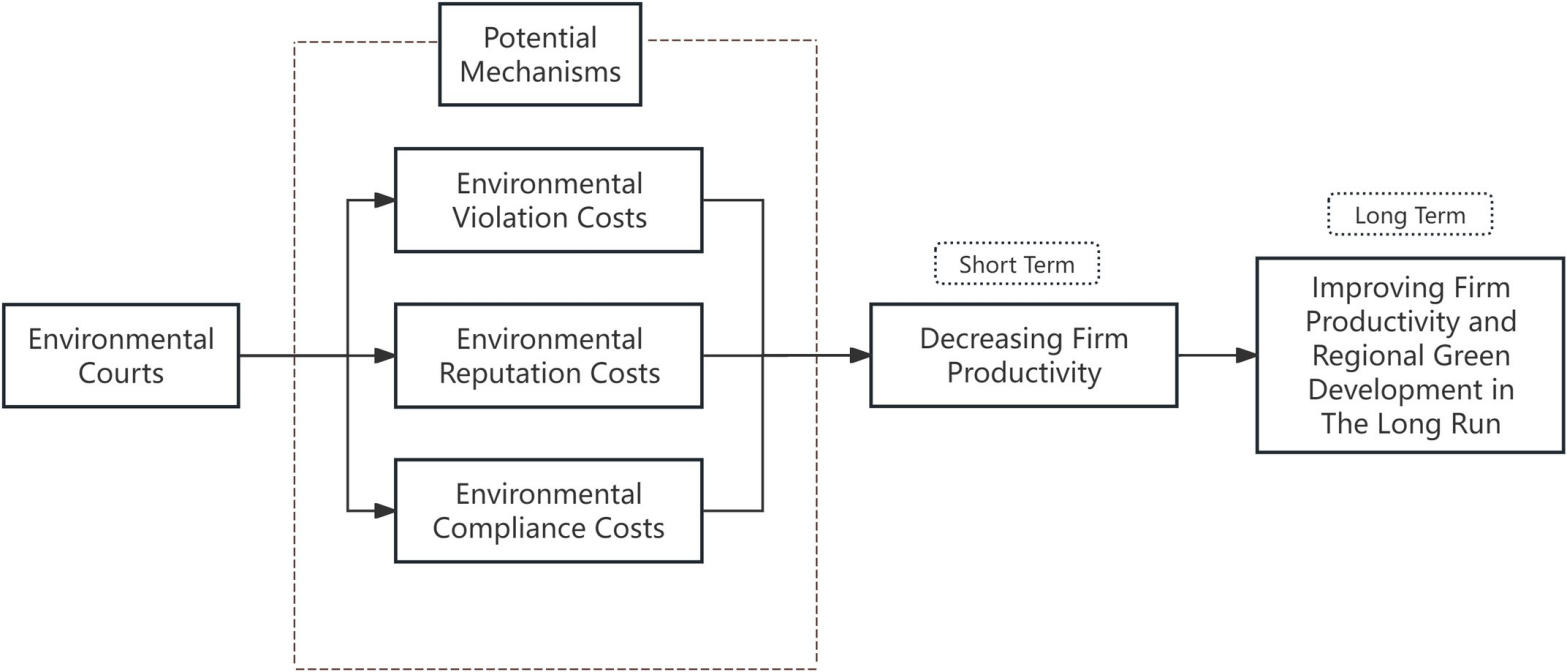
Framework.

In the long term, environmental courts may improve firm productivity. This is because, establishing environmental courts may reduce the short-sightedness of managers, induce firms to strengthen environmental protection through green innovations and transformation. As firm R&D and innovation is a time-consuming and risky activity, and it also takes time for firms to adjust and adapt to environmental justice, we anticipate that the value-enhancing effects and cost offsetting effects of the green transformation will become apparent in the long term. Thus, environmental courts can improve firms’ long-term productivity and regional green productivity. The theoretical analysis process is shown in Fig 2.

## 3. Data and identification strategy

### 3.1. Data and sample

We restrict our sample to A-share manufacturing firms in the Shanghai and Shenzhen stock markets from 2004 to 2014. The establishment of environmental courts provides an ideal research scenario to empirically examine how environmental judicature affect firm productivity. To eliminate potential confounding effects caused by the implementation of the "New Environmental Protection Law", we set the end year of the data to 2014. We screened the sample as follows: (1) removing the samples with an abnormal trading status (ST, PT and ST*). (2) eliminating the samples with missing variables.

The data are from several sources: (1) The data on the establishment time of the environmental courts are manually collected from the official websites of the Intermediate People’s Courts in China and other related news reports. (2) Firm financial information and media coverage data are obtained from the China Stock Market and Accounting Research Database. (3) Firm green innovation data is obtained from the China Research Data Services Platform. (4) The data on corporate environmental expenditure and environmental investment are from CCER database. (5) The data on ventilation coefficient and thermal inversion are from the European Centre for Medium-Term Weather Forecasting (ECMWF) ERA-Interim dataset and NASA, respectively. (6) The data on the number of local environmental cases accepted by the intermediate court are manually collected by the authors from the ITSLAW website. (7) We obtain the provincial government intervention index, and market-based intermediary development and legal institution environment index from the WIND database. (8) Other variables are obtained from the China City Statistical Yearbook. The main continuous variables are winsorized at the 1% level.

#### 3.1.1. Environmental court

We identify the exact year of establishment of environmental courts in prefecture-level cities in China for following reasons. On the one hand, four environmental courts have been established in provincial high people’s courts by 2014, which may underestimate the influence of the entire environmental courts. On the other hand, based on the availability of specific establishment time, we do not use the data on grassroots environmental courts.

#### 3.1.2. Firm productivity

We use the LP method to estimate *TFP* as a measure of firm productivity [31]. *TFP* indicates the driver of firm total output net of total input [32]. We use the Cobb-Douglas production function to portray firm production period, and a firm’s *TFP* is calculated by regression residual.


$$y_{it}=\alpha_{0}+\alpha_{k}k_{it}+\alpha_{l}l_{it}+\alpha_{m}m_{it}+\omega_{it}+\epsilon_{it}$$ (1)

Where *i*, *t* denotes firm and year respectively. *y*_*it*_ is firm output, measured by the natural logarithm of revenue; *k*_*it*_ is firm capital input, defined as the natural logarithm of fixed assets; *l*_*it*_ is firm labor input, measured by the natural logarithm of cash paid to and for employees; and *m*_*it*_ is firm intermediate inputs, defined as the natural logarithm of cash paid for goods purchased and services received. *ω_it_* indicates part of residual captures a firm’s production factors reallocation according to current production process.

#### 3.1.3. Control variables

According to the prior literature [33–35], we conclude city level and firm level variables as control variables. The control variables are as follows: (1) *GDP*, which refers to annual gross domestic product growth; (2) *FIA*, defined as fixed-asset investment scaled by gross domestic product; (3) *SED*, which refers to the proportion of secondary industry; (4) *FDI*, which is measured by the natural logarithm of the actual amount of foreign investment; (5) *SIZE*, which is measured as the natural logarithm of the book value of total assets; (6) *LEV*, defined as total liabilities divided by total assets; (7) *TQ*, which is measured by the sum of the market value of equity and liabilities divided by total assets; (8) *CF*, defined as net cash flow from operating activities divided by total assets; (9) *AGE*, defined as the number of years since a firm is established; (10) *BSIZE*, which is measured as the natural logarithm of the number of directors.

### 3.2. Empirical identification

As an exogenous event, the establishment of environmental courts provides us an optimal research scenario. Considering that DID method has been widely used for casual identification [6, 36, 37], we decide to use DID model to explore the effect of environmental courts on firm productivity. Due to the difference in the time of environmental courts establishment, we use the following multi-period DID model:

$$TFP_{ict}=\alpha +\beta \times EC_{ct}+\gamma \times Control_{ict-1}+\delta_{i}+\theta_{t}+\varepsilon_{ict}$$ (2)

Where *i*, *c*, *t* denotes firm, city, and year, respectively. The dependent variable, *TFP*_*ict*_, is measured by LP method. *EC*_*ct*_ equals to one if an environmental court is located in city *c* and in year *t*. Control is a vector of all control variables, including *GDP*, *FIA*, *SED*, *FDI*, *SIZE*, *LEV*, *TQ*, *CF*, *AGE*, and *BSIZE*, which are consistent with control variables section. We lag all control variables by one period to capture the delayed effects of them on *TFP*. Firm and year fixed effects are controlled in the model.

### 3.3. Statistical description

Our treatment group consists of 1811 firms located in cities with environmental courts until 2014, while 6281 firms located in cities without environmental courts serve as the control group. The dependent variable is firm productivity, represented by firms’ total factor productivity (*TFP*). The main independent variable is environmental courts (*EC*), defined as one if firms are situated in the cities that have set up environmental courts, and zero otherwise.

Table 1 shows the statistical description of our key variables. First, the maximum value and mean values of *TFP* in the treatment group are 9.64 and 7.269, respectively, while they are 9.64 and 7.268 in the control group, indicating that the *TFP* of firms in the treatment group does not vary significantly compared to the *TFP* in the control group. Second, the mean value of *EC* is 0.366 in the treatment group, indicating that 36.6% of the firm samples are located in the cities that have established environmental courts in treatment group. Besides, to eliminate the potential confounding effect of city characteristics and firm characteristics on *TFP*, our estimates include city-level control variables and firm-level control variables, which are comparable in both the treatment group and the control group in Table 1.

**Table 1:** The statistical description of key variables.

	Panel A: Treatment Group				Panel B: Control Group			
	Max	Mean	Min	N	Max	Mean	Min	N
Dependent Variable								
TFP	9.640	7.269	5.283	1811	9.640	7.268	5.283	6281
Main Independent Variable								
EC	1	0.366	0	1811	0	0	0	6281
City-level Controls								
GDP	0.383	0.162	0.015	1811	0.383	0.154	0.015	6281
FIA	1.073	0.571	0.233	1811	1.073	0.501	0.172	6281
SED	0.695	0.518	0.203	1811	0.695	0.479	0.203	6281
FDI	22.939	20.735	15.809	1811	23.444	20.536	15.809	6281
Firm-level Controls								
SIZE	24.908	21.471	19.538	1811	24.945	21.553	19.538	6281
LEV	0.851	0.447	0.050	1811	0.851	0.440	0.050	6281
TQ	6.532	1.681	0.926	1811	6.532	1.777	0.926	6281
CF	0.251	0.045	-0.150	1811	0.251	0.047	-0.150	6281
AGE	3.219	2.437	1.386	1811	3.219	2.459	1.386	6281
BSIZE	2.708	2.186	1.609	1811	2.708	2.192	1.609	6281

Note: Table 1 reports the summary statistics of main variables. All variables are defined in data and sample section as above.

## 4. Results

### 4.1. Baseline results

Table 2 shows the results of the relationship between environmental courts and firm productivity by employing multi-period DID strategy. First, since firm characteristics may affect firm business strategy and environmental behavior, we include firm characteristics and firm fixed effect to capture the time-varying and time-unvarying confounding effects at firm level. Column 1 shows that the coefficient of *EC* is -0.076 and significant at 1% level, which suggests that environmental courts inhibit firm productivity in the short term. Second, to eliminate the impact of other time-varying policies, Model (2) further include year fixed effect, and Column 2 shows the result. The coefficient of *EC* is -0.045 and significant.

**Table 2:** Environmental courts and firm productivity.

	(1)	(2)	(3)	(4)
	TFP	TFP	TFP	TFP
EC	-0.076***	-0.045**	-0.075***	-0.045**
	(-3.713)	(-2.147)	(-3.640)	(-2.149)
GDP			0.193***	0.170*
			(2.827)	(1.946)
FIA			0.092	0.020
			(1.251)	(0.257)
SED			-0.202	-0.123
			(-1.037)	(-0.617)
FDI			0.015	0.009
			(0.993)	(0.594)
SIZE	0.380***	0.381***	0.378***	0.381***
	(17.927)	(17.999)	(18.205)	(18.096)
LEV	0.275***	0.250***	0.273***	0.249***
	(3.465)	(3.131)	(3.442)	(3.118)
TQ	0.072***	0.058***	0.069***	0.057***
	(10.539)	(6.865)	(10.354)	(6.817)
CF	0.354***	0.346***	0.373***	0.348***
	(5.271)	(5.157)	(5.538)	(5.192)
AGE	0.069*	-0.115	0.040	-0.119
	(1.829)	(-1.253)	(0.942)	(-1.312)
BSIZE	-0.047	-0.032	-0.048	-0.034
	(-0.754)	(-0.527)	(-0.766)	(-0.555)
Constant	-1.239***	-0.809*	-1.389***	-0.954*
	(-3.229)	(-1.845)	(-3.436)	(-1.939)
Firm FE	YES	YES	YES	YES
Year FE	NO	YES	NO	YES
N	8092	8092	8092	8092
Adj.R^2^	0.900	0.903	0.900	0.903

Note: Table 2 reports the effect of environmental courts on firm productivity based on a multi-period DID model. Robust t-statistics clustered at the firm level are reported in parentheses. ^*^, ^**^, and ^***^ denote significance at the 10%, 5%, and 1% levels, respectively.

Besides, city characteristics also have an important impact on firm productivity. Thus, we further control for city-level variables. Column 3 presents the result when Model (2) includes all control variables and firm fixed effect, and our conclusion still holds. Column 4 further includes year fixed effect. The coefficient of *EC* is significantly negative, indicating a 4.5% productivity loss for those firms located in cities have established environmental courts because of higher environmental cost burden in the short term.

### 4.2. Endogeneity analysis

#### 4.2.1. Parallel trend

To increase the robustness of multi-period DID regression results, we further examine whether firm productivity between the treatment group and control group meet the parallel trend before the establishment of environmental courts. Thus, we construct model (3) as follows [38, 39]:

$$TFP_{ict}=\alpha +\beta_{1}^{\prime }\times \sum_{\rho =2}^{4}\;Before_{ict}^{x}+\beta_{2}^{\prime }\times \sum_{\lambda =0}^{4}\;After_{ict}^{k}+\gamma \times Control_{i(c)t-1}+\delta_{i}+\lambda_{t}+\varepsilon_{ict}$$ (3)

$Before_{ict}^{x}$takes a value of 1 if firms are situated in cities where the environmental court was set up *x* years prior; otherwise, it is 0. $After_{ict}^{k}$takes a value of 1 if firms are situated in cities where the environmental court was set up *k* years afterward; otherwise, it is 0. Due to low sample sizes in pre-treatment periods 5–10 and post-treatment periods 5–6, we set *x* = 4 if *x*> = 4 and set *k* = 4 if *k*> = 4. We select Before 1 as the base year.

**Fig 3:**
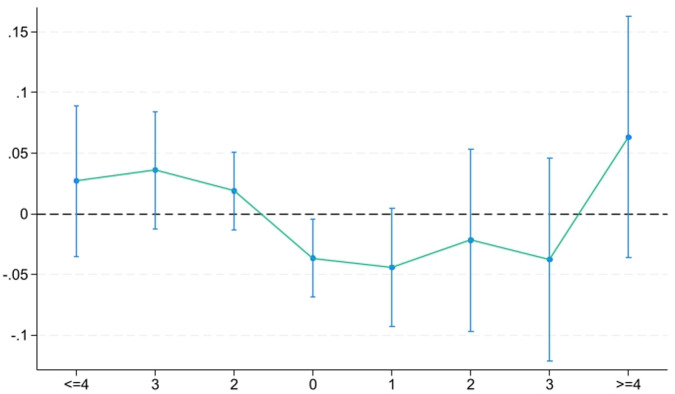
The Parallel trend test.

Fig 3 depicts the results of above tests. It shows that *Before*^*4*^, *Before*^*3*^ and *Before*^*2*^ are insignificant, suggesting that the parallel trend assumption is satisfied. Second, *After*^*0*^ is significantly negative, which provides evidence that environmental courts inhibit firm productivity in the short run. Third, *After*^*1*^, *After*^*2*^, *After*^*3*^ and *After*^*4*^ are insignificant, suggesting the inhibiting impact of environmental courts on firm productivity would be weakened in the long term.

#### 4.2.2. Mitigation of heterogeneous treatment effects

Recent studies have shown that TWFE regressions of multi-period DID may suffer from negative weights due to heterogeneous treatment effects, which leads to biased baseline results [40, 41]. Using the method proposed by De Chaisemartin and d’Haultfoeuille [41], we test the negative weights problem of regression results. After decomposing the weights in the DID regressions, it was revealed that there are 61 groups with negative weights and 602 groups with positive weights, with negative weights constituting only 9.2 percent of the total. This suggests that our baseline results are robust.

Although the test of negative weights problem has demonstrated that our baseline results are minimally influenced by the negative weight problem, the paper also employs the stacked DID method to re-estimate for mitigating heterogeneous treatment effects [42]. Table 3 represents the result. The average treatment effect is still significantly negative, further showing that our conclusions are robust.

**Table 3:** Heterogeneity-robust DID.

	Coef.	Std. Err.	t	P>|t|
ATT	-0.128	0.073	-1.76	0.079

Note: “ATT” means Average Treatment Effect for the Treated Group. Table 3 shows the coefficient, standard errors, t value and related p value of the average treatment effect.

**Fig 4:**
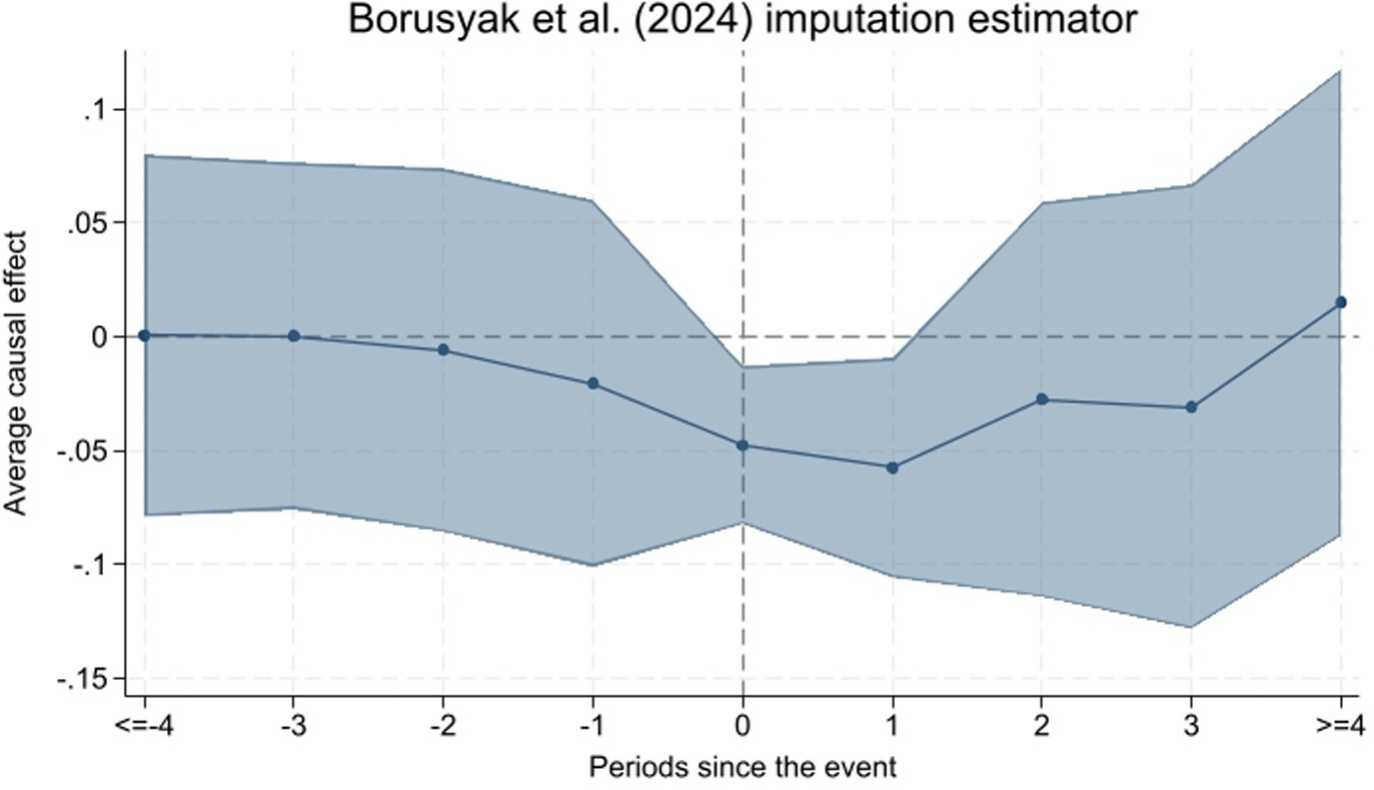
Borusyak et al. (2024) imputation estimator.

We further use event study method generated heterogeneity-robust estimator to verify our main findings [43]. As Fig 4 shown, the coefficients of before current period are not significant, while the coefficients of the current period and the first period are significant, indicating that our conclusions are robust.

#### 4.2.3. PSM-DID estimation

To mitigate the interference of problem of sample self-selection, this paper uses the propensity score matching (PSM) method. Taking firm characteristics such as *SIZE*, *LEV*, *TQ*, *CF*, *AGE*, *BSIZE* and city characteristics such as *GDP*, *FIA*, *SED*, *FDI* as control variables, we use the 1:2 proximity matching approach to formulate the right control group. Using the matched sample, we further perform DID estimates.

PSM-DID estimation presents in Table 4. Column 1 reports the regression result without controlling for year fixed effect. The coefficient of *EC* is significantly negative. Column 2 further includes year fixed effect, and the result remains unchanged, suggesting that environmental courts reduce firm productivity in the short run after accounting the endogenous interference of sample bias.

**Table 4:** PSM-DID estimation and IV estimation.

	(1)	(2)	(3)	(4)
	TFP	TFP	EC	TFP
			First Stage	Second Stage
EC	-0.095**	-0.084**		-0.588*
	(-2.463)	(-2.029)		(-1.660)
VC			-0.046***	
			(-3.053)	
TID			0.519***	
			(2.760)	
GDP	0.271	0.401	-0.033	0.154*
	(1.069)	(1.262)	(-0.833)	(1.889)
FIA	0.261	0.246	0.151***	0.107
	(1.315)	(1.103)	(5.649)	(1.395)
SED	-0.313	-0.154	-0.570***	-0.431*
	(-0.561)	(-0.256)	(-6.657)	(-1.735)
FDI	0.125**	0.129**	-0.015***	0.001
	(2.043)	(2.088)	(-2.707)	(0.076)
SIZE	0.192***	0.192***	0.020***	0.393***
	(3.760)	(4.443)	(2.631)	(22.580)
LEV	0.025	0.015	0.024	0.261***
	(0.147)	(0.092)	(0.875)	(4.490)
TQ	0.015	0.006	-0.000	0.057***
	(0.539)	(0.209)	(-0.035)	(7.361)
CF	0.084	0.064	0.068*	0.385***
	(0.370)	(0.266)	(1.738)	(5.258)
AGE	0.261*	0.401	0.084**	-0.077
	(1.738)	(1.027)	(2.465)	(-1.185)
BSIZE	0.329***	0.339***	0.042**	-0.012
	(4.291)	(4.446)	(2.141)	(-0.285)
Constant	-0.796	-1.307		
	(-0.602)	(-0.837)		
FIRM	YES	YES	YES	YES
YEAR	NO	YES	YES	YES
N	7393	7393	8088	8088
Adj.R^2^	0.960	0.960		0.335
F			10.965	
Hansen J statistic				0.110
P				0.740

Note: Table 4 shows the results of PSM-DID estimation and IV estimation. Robust t-statistics clustered at the firm level are reported in parentheses in Columns 1–3, and z-statistics are reported in parentheses in Column 4. ^*^, ^**^, and ^***^ denote significance at the 10%, 5%, and 1% levels, respectively.

#### 4.2.4. IV estimation

A potential issue with the baseline result is that the location and timing of environmental courts could be influenced by other factors, which may lead to endogenous problem. Therefore, we utilize an instrumental variable (IV) strategy to mitigate this concern [8, 27, 44]. Our instrumental variables consist of the logarithm of the ventilation coefficient (*VC*) and the frequency of days with thermal inversion in the first and third layers (*TID*) per year in the city level.

Following Hering and Poncet [45], Cai, Lu [46] and Shi and Xu [47], we calculate the ventilation coefficient by the wind speed at a height of 10 meters multiplied by the height of the boundary layer. The European Centre for Medium-Term Weather Forecasting (ECMWF) ERA-Interim dataset provides the data on the wind speed and the height of the boundary layer for the grid of 75×75 cells. We match the latitude and longitude of each city with the dataset and select the ventilation coefficient of the nearest grid cell as the ventilation coefficient for each city. According to Chen, Oliva [48], we further use the frequency of days with thermal inversion in the first and third layers per year in the city level as our instrumental variable. Typically, air temperature decreases with rising altitude, leading to vertical air convection. However, thermal inversion represents the opposite scenario, where higher altitudes correspond to higher temperatures. This condition hampers vertical air convection and results in the aggregation of air pollution. The NASA provides global temperatures at different altitudes at six-hour intervals. We initially computed the daily average temperatures separately for each city at the third level (540 meters) and the first level (110 meters). If the average temperature at the third level was higher than that at the first level, we considered it as thermal inversion. On the one hand, both instrumental variables are significantly related to local pollution and satisfy the requirement of correlation. Since local pollution levels tend to be higher in cities with lower ventilation coefficients and a higher frequency of thermal inversions, which increases the likelihood of establishing environmental courts. On the other hand, as meteorological indicators, both ventilation coefficients and the frequency of days with thermal inversions show no direct correlation with firm productivity, thus meeting the exogeneity criterion.

Table 4 reports the results of instrumental variable approach. In Column 3, the coefficient of *VC* is -0.046, which has a negative correlation with the likelihood of environmental court establishment. The coefficient of *TID* is 0.519, which positively correlates with the likelihood of establishing an environmental court. The F-value of the first stage regression exceeds 10, indicating that there is no problem with weak instrumental variables. In Column 4, the coefficient of *EC* is significantly negative. The P-value of the Hansen J statistic is 0.74, validating the exogeneity of instrumental variables. The IV estimation indicates that our conclusion is robust after mitigating the endogeneity problem.

#### 4.2.5. Placebo tests

**Fig 5:**
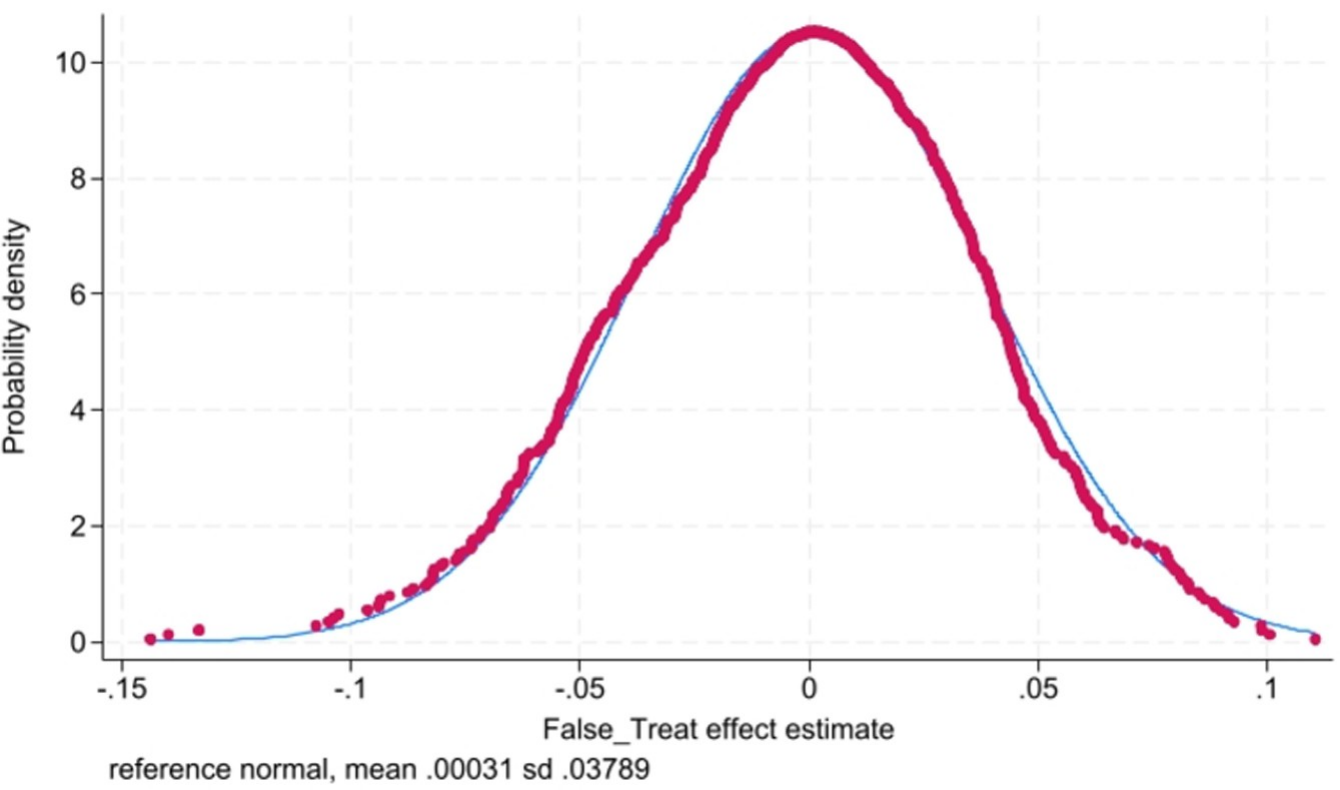
Bootstrapping method.

The empirical regression results in this paper may be random, rather than the real effect. Thus, following the existing literature [46], we use bootstrapping method to obtain the list of false cities where environmental courts have been established and false establishment times by randomly sampling from the city-year sample, and match this list to firm-level data, then run the model (2) to obtain the false coefficient of environmental courts (*False_Treat*). The above process is repeated 1000 times. According to *False_Treat*, Fig 5 could be obtained. The symmetry axis is close to 0, which indicates there is a real causal relationship between environmental courts and firm productivity.

### 4.3. Additional robustness checks

#### 4.3.1. Changing the sample period

Sample period may affect the effect of environmental courts. Therefore, we reconstruct our regression sample from 2003 to 2014 to re-examine the result. According to Column 1 of Table 5, we observe that the coefficient of *EC* is significantly negative. The result remains robust.

**Table 5:** Additional robustness checks (I).

	(1)	(2)	(3)	(4)
	TFP2	TFP	TFP	TFP3
EC	-0.045**	-0.076***	-0.057**	-0.035*
	(-2.120)	(-2.631)	(-2.431)	(-1.680)
GDP	0.161*	0.257	0.063	0.096
	(1.795)	(1.275)	(0.838)	(1.127)
FIA	0.037	-0.093	0.032	-0.013
	(0.466)	(-0.592)	(0.424)	(-0.188)
SED	-0.178	0.511	0.002	-0.123
	(-0.852)	(1.093)	(0.009)	(-0.637)
FDI	0.014	-0.009	0.020	0.009
	(0.943)	(-0.227)	(1.436)	(0.576)
SIZE	0.381***	0.381***	0.343***	0.110***
	(18.419)	(7.575)	(16.892)	(5.403)
LEV	0.254***	0.372***	0.275***	0.304***
	(3.052)	(2.817)	(3.722)	(3.993)
TQ	0.059***	0.077***	0.052***	0.032***
	(6.898)	(3.760)	(6.242)	(4.251)
CF	0.371***	0.297**	0.284***	0.135**
	(5.502)	(2.250)	(4.472)	(2.166)
AGE	-0.155*	-0.033	-0.111	-0.147
	(-1.772)	(-0.180)	(-1.166)	(-1.605)
BSIZE	-0.013	0.020	0.000	-0.068
	(-0.211)	(0.176)	(0.007)	(-1.243)
Constant	-1.119**	-1.231	-0.502	0.876*
	(-2.293)	(-1.264)	(-0.945)	(1.720)
FIRM	YES	YES	YES	YES
YEAR	YES	YES	YES	YES
CITY*YEAR	NO	NO	YES	NO
PRO*YEAR	NO	NO	YES	NO
IND*YEAR	NO	NO	YES	NO
N	8526	1811	8092	8092
Adj.R^2^	0.893	0.901	0.912	0.809

Note: Table 5 presents the results of additional robustness checks (I). Robust t-statistics clustered at the firm level are reported in parentheses. ^*^, ^**^, and ^***^ denote significance at the 10%, 5%, and 1% levels, respectively.

#### 4.3.2. Removing samples of firms located in cities without environmental courts during 2004–2014

Some prefecture-level cities have never established environmental courts between 2004–2014, which may lead to too many zero values of *EC* and make the regression results biased. To alleviate this concern, we remove the samples of firms located in cities without environmental courts during 2004–2014 and then perform the DID strategy. We can find that environmental courts reduces firm productivity from Column 2 of Table 5, indicating the result we obtain is robust.

#### 4.3.3. Controlling other policy shocks

Considering that some unobserved city-level, provincial level and industry level confounding factors might influence firm productivity, leading to varying productivity trends in different cities, provinces and industries. To exclude the linear time trend, we further control the interactions between *CITY×YEAR*, *PRO×YEAR* and *IND×YEAR* to re-estimate the result. The results are reported in Column 3 of Table 5. The coefficients of *EC* remain significant.

#### 4.3.4. Rebuilding the measurement of firm productivity

Levinsohn–Petrin (LP) method and the Olley-Pakes (OP) method are widely used methods to calculate TFP [31, 49]. In baseline part, we employ the Levinsohn–Petrin (LP) method to calculate *TFP* as a proxy for firm productivity [31].To mitigate metric bias, we employ OP method to rebuild variable *TFP3*. Column 4 of Table 5 reports the result. The coefficient of *EC* is still within our expectations, indicating the baseline results are robust.

#### 4.3.5. Excluding samples of municipalities

Considering the unique aspects of municipalities directly under the central government in terms of economic development and administrative levels, regressing the samples of municipalities and other prefecture-level cities together may lead to a biased regression result. Thus, we exclude the samples of municipalities, and report the result in Column 1 of Table 6. It shows that environmental courts decrease firm productivity in the short term.

**Table 6:** Additional robustness checks (II).

	(1)	(2)	(3)	(4)
	TFP	TFP	TFP	TFP
EC	-0.044**	-0.048**	-0.046*	-0.045**
	(-2.062)	(-2.233)	(-1.932)	(-2.085)
GDP	0.204**	0.169*	0.176*	0.170*
	(2.253)	(1.902)	(1.897)	(1.932)
FIA	0.021	0.022	0.017	0.020
	(0.249)	(0.274)	(0.217)	(0.269)
SED	-0.054	-0.117	-0.102	-0.123
	(-0.255)	(-0.586)	(-0.506)	(-0.573)
FDI	0.012	0.005	0.004	0.009
	(0.749)	(0.337)	(0.237)	(0.659)
SIZE	0.384***	0.384***	0.381***	0.381***
	(16.498)	(18.740)	(18.206)	(17.863)
LEV	0.214**	0.255***	0.246***	0.249***
	(2.496)	(3.162)	(2.928)	(2.903)
TQ	0.056***	0.057***	0.056***	0.057***
	(5.945)	(5.957)	(6.555)	(6.973)
CF	0.317***	0.387***	0.326***	0.348***
	(4.513)	(5.446)	(4.312)	(5.754)
AGE	-0.158*	-0.127	-0.109	-0.119
	(-1.684)	(-1.382)	(-1.199)	(-1.349)
BSIZE	-0.032	-0.039	-0.007	-0.034
	(-0.479)	(-0.614)	(-0.110)	(-0.597)
Constant	-1.003*	-0.933*	-0.925*	-0.954**
	(-1.913)	(-1.861)	(-1.876)	(-2.011)
FIRM	YES	YES	YES	YES
YEAR	YES	YES	YES	YES
N	6998	7492	7033	8092
Adj.R^2^	0.903	0.904	0.899	0.903

Note: Table 6 presents the results of additional robustness checks (II). Robust t-statistics clustered at the firm level are reported in parentheses. ^*^, ^**^, and ^***^ denote significance at the 10%, 5%, and 1% levels, respectively.

#### 4.3.6. Excluding the confounding effect of financial crisis

Our sample period is 2003–2014, including the financial crisis in 2008. It may reduce firm productivity, resulting in an upward bias of the inhibitory effect of environmental courts obtained in this paper. Thus, we drop samples in 2008 and re-estimate. In Column 2 of Table 6, it shows that the baseline results still hold.

#### 4.3.7. Excluding the confounding effect of green credit guidelines

Firms’ credit availability affects firms’ financing constraints and hence firms’ productivity. The green credit guidelines issued in 2012 affect access to credit for firms with different pollution intensities, which may influence firm productivity. To eliminate the potential influence, the paper further excludes the samples in 2012, and the result is presented in Column 3 of Table 6. It is as expected.

#### 4.3.8. Changing the cluster level

In the previous empirical results, t-statistics are clustered at the firm level based on the assumption that the observations of a firm at different times are correlated, but different firms are independent. Considering that various clustering levels could impact the significance of the regression, we further cluster the t-statistics at the city level. The result in Column 4 of Table 6 shows the robustness of the baseline results.

### 4.4. Potential mechanisms

#### 4.4.1. Environmental violation costs

Environmental courts may decrease firm productivity through the environmental violation costs channel. First, while environmental courts offer a convenient avenue for victims of environmental pollution to seek legal redress, they also increase the likelihood of firms facing environmental lawsuits. Second, higher judicial efficiency and stricter judicial enforcement lead to higher financial losses for firms involved in environmental cases. Therefore, firms are expected to bear higher environmental violation costs after the establishment of environmental courts. Considering firm environmental violation costs are associated with the number of local environmental cases, we measure environmental violation costs (*EVC*) as the natural logarithm of one plus the number of environmental cases accepted by the intermediate court in city *c* in year *t*. Since *EVC* is a city-level variable, we construct model (4) to test the relationship between environmental courts and EVC.


$$EVC_{ct}=\alpha +\beta \times EC_{ct}+\gamma \times Control_{ct-1}+\mu_{c}+\theta_{t}+\varepsilon_{ict}$$ (4)

Where *c*, *t* denotes city and year, respectively. The dependent variable is *EVC*_*ct*_. *EC*_*ct*_ is independent variable. Control is a vector of all control variables, including *GDP*, *FIA*, *SED* and *FDI*. Table 7 reports the results of the mechanism tests. Columns 1–2 report the relationship between environmental courts and firm environmental violation costs. In Column 1, the coefficient of *EC* is 0.215 and is statistically significant at the 10% level. Column 2 reports the result of further controlling for city characteristics. *EC* is 0.205, and significant at the 10% level, confirming the environmental violation costs channel.

**Table 7:** Potential mechanisms.

	(1)	(2)	(3)	(4)	(5)	(6)
	EVC	EVC	ERC	ERC	ECC	ECC
EC	0.215*	0.205*	0.060**	0.059**	0.059*	0.059*
	(1.899)	(1.854)	(2.383)	(2.340)	(1.944)	(1.915)
GDP		0.139*		0.043		0.045
		(1.706)		(0.301)		(0.476)
FIA		-0.365***		0.015		0.071
		(-4.785)		(0.125)		(1.155)
SED		-0.003		0.171		0.039
		(-1.277)		(0.441)		(0.175)
FDI		-0.035***		-0.041		-0.003
		(-2.826)		(-1.376)		(-0.180)
SIZE			0.030	0.029	0.007	0.007
			(1.492)	(1.422)	(0.409)	(0.382)
LEV			0.039	0.045	0.257***	0.253***
			(0.355)	(0.417)	(3.969)	(3.927)
TQ			0.012	0.012	-0.017**	-0.018**
			(1.406)	(1.333)	(-2.074)	(-2.101)
CF			-0.083	-0.091	0.043	0.046
			(-0.979)	(-1.073)	(0.749)	(0.796)
AGE			-0.022	-0.005	-0.109	-0.101
			(-0.177)	(-0.039)	(-1.217)	(-1.122)
BSIZE			0.076	0.076	0.007	0.006
			(0.775)	(0.768)	(0.112)	(0.102)
Constant	0.009	0.879***	-0.717	0.017	0.099	0.104
	(0.611)	(3.347)	(-1.431)	(0.027)	(0.220)	(0.180)
FIRM	NO	NO	YES	YES	YES	YES
YEAR	YES	YES	YES	YES	YES	YES
CITY	YES	YES	NO	NO	NO	NO
N	2985	2985	8092	8092	8092	8092
Adj.R^2^	0.414	0.427	0.314	0.314	0.353	0.353

Note: Table 7 shows the results of three potential mechanisms between environmental courts and firm productivity. Columns 1–2 show the results of the city panel data regression, and Columns 3–6 show the the result of the firm panel data regression. Robust t-statistics clustered at the city level or firm level are reported in parentheses. ^*^, ^**^, and ^***^ denote significance at the 10%, 5%, and 1% levels, respectively.

#### 4.4.2. Environmental reputation costs

Environmental reputation costs may also be a potential influence mechanism of environmental courts affecting firm productivity. By enhancing public attention and scrutiny of environmental issues, environmental courts can damage a firm’s reputation and image, thereby increasing environmental reputation costs. This heightened scrutiny raises the perceived risk for a firm’s stakeholders, which not only increases the cost of financing, but also reduces the market share of sales and undermines supply chain stability [28–30]. Thus, the rising environmental reputation costs reduce firm productivity. Given the difficulty of directly measuring environmental reputation costs, we use the ratio of media reports on a firm’s environmental problems to its total media coverage as a proxy. This approach assumes that greater media attention to a firm’s environmental issues leads to stronger public scrutiny and, consequently, higher environmental reputation costs (*ERC*).

Columns 3–4 of Table 7 report the relationship between environmental courts and firm environmental reputation costs. Column 3 reports the results after controlling for firm fixed effect, year fixed effect and firm-level control variables. The coefficient of *EC* is 0.06, and statistically significant at the 5% level. Column 4 reports the results after controlling for firm fixed effect, year fixed effect and all control variables. The coefficient of *EC* is 0.059, and remains significant at the 5% level, confirming the firm environmental reputation costs mechanism.

#### 4.4.3. Environmental compliance costs

Environmental courts may also decrease firm productivity through the environmental compliance costs channel. Environmental courts will increase firms’ expenditures and investment in environmental protection. On the one hand, it may reduce the firms’ cash outflow and increase financial pressure. On the other hand, due to a firm’s resource limitation, when a firm enhances environmental investment, it may decrease productive investment, which will decrease firm productivity. To verify the mechanism, we use the sum of fee expenditures and capital expenditure on environmental protection scaled by owners’ equity to measure firm environmental compliance costs (*ECC*).

Columns 5–6 of Table 7 report the relationship between environmental courts and firm environmental compliance costs. Column 5 reports the results after controlling for firm fixed effect, year fixed effect and firm-level control variables. The coefficient of *EC* is 0.06, and statistically significant at the 10% level. Column 6 reports the results after controlling for firm fixed effect, year fixed effect and all control variables. The coefficient of *EC* is 0.059, and remains significant at the 10% level, confirming the firm environmental compliance costs channel.

### 4.5. Heterogeneity analysis

#### 4.5.1. Legal situation

The influence of environmental courts on firm productivity would be affected by local legal conditions. Regions with greater legal authority would raise the environmental cost burden for firms, resulting in reduced firm productivity after environmental courts establishment. This inhibition would be more significant in regions with high legal authority compared to those with low legal authority. Based on the above analysis, according to the annual industry median of the market-based intermediary development and legal institution environment index in province level, we separate the sample across two parts: firms in regions with high legal scores (*HLS*) and firms in regions with low legal scores (*LLS*). Column 1–2 of Table 8 report the results for the two sub-samples, respectively. We observe that environmental courts significantly reduce firm productivity only in the sub-samples with high legal scores, but not in those with low legal scores. The Fisher’s Permutation Test provides further evidence of the robustness of the conclusions. This may be because regions with high legal scores pay more attention to case trial and law enforcement, which can better play the role of environmental courts. This leads to higher environmental cost burden for firms, which in turn inhibits firm productivity.

**Table 8:** Heterogeneity analysis (I).

	(1)	(2)	(3)	(4)
	HLS	LLS	LGI	HGI
	TFP	TFP	TFP	TFP
EC	-0.077***	-0.007	-0.088***	0.022
	(-4.180)	(-0.168)	(-3.487)	(0.616)
GDP	-0.051	0.241*	0.066	0.088
	(-0.395)	(1.972)	(0.620)	(0.705)
FIA	-0.094	0.079	-0.113	0.032
	(-0.811)	(0.723)	(-0.959)	(0.300)
SED	0.020	-0.102	-0.125	-0.172
	(0.068)	(-0.333)	(-0.422)	(-0.646)
FDI	0.017	0.005	0.027	-0.010
	(0.500)	(0.386)	(1.333)	(-0.611)
SIZE	0.376***	0.389***	0.368***	0.387***
	(13.021)	(11.936)	(12.209)	(13.012)
LEV	0.315***	0.195	0.334***	0.235**
	(2.906)	(1.601)	(3.271)	(1.987)
TQ	0.058***	0.056***	0.054***	0.061***
	(6.238)	(3.976)	(5.541)	(4.410)
CF	0.361***	0.330***	0.377***	0.308***
	(4.589)	(3.638)	(4.307)	(2.909)
AGE	-0.172*	-0.187	-0.125	-0.231*
	(-1.762)	(-1.202)	(-1.037)	(-1.692)
BSIZE	-0.062	-0.058	-0.051	-0.084
	(-1.281)	(-0.593)	(-0.844)	(-0.885)
Constant	-0.808	-0.897	-0.926	-0.309
	(-0.879)	(-1.165)	(-1.203)	(-0.500)
FIRM	YES	YES	YES	YES
YEAR	YES	YES	YES	YES
N	4061	3839	3851	3951
Adj.R^2^	0.905	0.905	0.909	0.907
Test	0.07**		0.109***	

Note: Table 8 reports the results of heterogeneity analysis. Robust t-statistics clustered at the firm level are reported in parentheses. ^*^, ^**^, and ^***^ denote significance at the 10%, 5%, and 1% levels, respectively. Test represents Fisher’s Permutation Test.

#### 4.5.2. Government intervention

Government intervention can hinder the effective role of environmental administration enforcement. Local governments have greater incentives to favor high-polluting firms due to their considerable contribution to local economic development, which has long been the primary criterion for the promotion of government officials. They can intervene in the process of administrative supervision. Environmental courts employ the cross-regional jurisdiction model, which helps to maintain judicial independence and mitigate the intervention of local government. It is anticipated that environmental courts could be more effective in regions with high government intervention, thereby increasing firms’ environmental cost burden and inhibiting their productivity. Thus, according to the annual industry median of the government intervention index in province level, we separate the sample across two parts: firms in regions with high government intervention (*HGI*) and firms in regions with low government intervention (*LGI*). From Column 3–4 of Table 8, it shows that the coefficient of *EC* is only significant in the sub-samples with low government intervention. The Fisher’s Permutation Test reveals a significant difference between the coefficients of *EC* in Column 3 and Column 4. This conclusion contradicts the findings of a previous study [6]. Our empirical evidence indicates that environmental courts are not completely free from government intervention.

#### 4.5.3. Geographic location

The geographical location of firms will also influence the impact of environmental courts on firm productivity. Given the economic and social development context for firms in the eastern and western regions varies significantly, we further divide our sample into two parts: *East* and *West*. Then, we employ the same DID strategy to re-estimate the results for each of the two sub-samples. In Column 1–2 of Table 9, *EC* is only significantly negative for firms in the eastern region. The Fisher’s Permutation Test demonstrates that the coefficients of *EC* in Column 1 and Column 2 differ significantly. This may be because the eastern region pays more attention to the legal institution establishment, and environmental courts play a more effective role in environmental judicature. In addition, environmental courts are more frequently located in the eastern region, which is also an important reason for this heterogeneous result.

**Table 9:** Heterogeneity analysis (II).

	(1)	(2)	(3)	(4)
	WEST	EAST	SMP	WMP
	TFP	TFP	TFP	TFP
EC	0.035	-0.076***	-0.013	-0.083***
	(0.743)	(-3.399)	(-0.443)	(-2.877)
GDP	0.286**	-0.084	0.217*	0.068
	(2.333)	(-0.637)	(1.921)	(0.541)
FIA	0.112	-0.219*	-0.032	0.151
	(0.938)	(-1.933)	(-0.354)	(1.155)
SED	-0.143	0.232	-0.006	-0.169
	(-0.484)	(0.724)	(-0.019)	(-0.603)
FDI	0.007	0.005	0.022	-0.013
	(0.359)	(0.230)	(1.110)	(-0.554)
SIZE	0.379***	0.379***	0.324***	0.397***
	(11.459)	(14.266)	(10.464)	(11.163)
LEV	0.233*	0.260**	0.240**	0.180
	(1.825)	(2.555)	(2.429)	(1.574)
TQ	0.050***	0.061***	0.049***	0.070***
	(3.301)	(6.471)	(5.606)	(3.758)
CF	0.343***	0.340***	0.268**	0.290***
	(3.372)	(3.953)	(2.312)	(3.371)
AGE	-0.194	-0.098	-0.153	-0.045
	(-1.194)	(-0.920)	(-1.389)	(-0.295)
BSIZE	0.012	-0.092	0.011	-0.104
	(0.125)	(-1.350)	(0.179)	(-1.101)
Constant	-0.885	-0.771	-0.196	-0.722
	(-1.192)	(-1.013)	(-0.263)	(-0.987)
FIRM	YES	YES	YES	YES
YEAR	YES	YES	YES	YES
N	3207	4885	3823	3992
Adj.R^2^	0.900	0.907	0.921	0.894
Test	0.11***		-0.07**	

Note: Table 9 reports the results of heterogeneity analysis. Robust t-statistics clustered at the firm level are reported in parentheses. *, **, and *** denote significance at the 10%, 5%, and 1% levels, respectively. Test represents Fisher’s Permutation Test.

#### 4.5.4. Firm market power

Firms’ ability to absorb the environmental cost shock may also influence the effectiveness of environmental courts. When firms have strong market power, they can pass on the cost burden to consumers by increasing the price of their products. At this point, firms face fewer costs shocks induced by environmental judicature. Firms with weak market power can not bear and absorb cost burdens, thus reducing firm productivity. We anticipate the influence of environmental courts to be greater in firms with weaker market power. We utilize the Lerner Index to assess firms’ market power [50], a metric that captures a firm’s ability to pass on costs. Lerner index is calculated as operating revenue minus operating costs divided by operating revenue [51]. Based on the industry annual median of the firm Lerner index, we split our sample across two sub-samples: firms with strong market power (*SMP*) and firms with weak market power (*WMP*). In Columns 3–4 of Table 9, it can be observed that the inhibitory effect of environmental courts on firm productivity is only significant for firms with weak market power, which confirms our reasoning. The Fisher’s Permutation Test reinforces the finding.

#### 4.5.5. Firm pollution intensity

The influence of environmental courts on firm productivity should be influenced by firm pollution intensity. Firms with high pollution intensity will have a higher likelihood of being prosecuted by environmental courts and of being subject to public scrutiny, and will therefore bear a greater cost of environmental violations and reputation. Firms with high pollution intensity also have greater environmental compliance pressures, leading to greater environmental compliance costs. These cost burden will reduce firm productivity. Conversely, firms with low pollution intensity do better with environmental compliance and are less exposed to environmental cost shocks. Thus, we anticipate that environmental courts have a more significant impact on firm productivity in samples characterized by high pollution intensity. Therefore, we divided the sample into two sub-groups: high pollution intensity enterprises (*HPI*) and low pollution intensity enterprises (*LPI*). First, we determine the pollution intensity of a firm based on its industry attributes. According to the Listed Companies Environmental Verification Industry Classification Management Directory issued by the Ministry of Ecology and Environment of the People’s Republic of China, if the sector of the sample is located in C15, C18, C17, C19, C22, C25, C26, C27, C28, C29, C30, C31, C32 and C33, then the enterprise is categorised as having a high environmental pollution intensity. Otherwise, it is a firm with low environmental pollution intensity. Second, we assess a firm’s pollution intensity by comparing its wastewater discharge to the industry annual median of wastewater discharge. Third, we evaluate a firm’s pollution intensity by comparing its sulfur dioxide emissions to the industry annual median of SO_2_ emissions. Since obtaining pollutant emission data at the firm level is difficult, we calculate the pollution emissions for each firm by using the ratio of the number of employees in the firm to total urban employment as weights. These weights are then applied to the municipal-level industrial wastewater and SO_2_ emission data to estimate the firm’s pollution emissions.

Columns 1–2 of Table 10 shows the results of the heterogeneity analysis of pollution intensity by industry attributes. It shows that *EC* is only significantly negative for firms with high pollution intensity, indicating that environmental courts result in increased cost burden of firms with high pollution intensity, thereby reducing their productivity. The Fisher’s Permutation Test provides additional confirmation of the finding. Columns 3–4 of Table 10 shows the results of the heterogeneity analysis by grouping firms by wastewater discharge. *EC* is only significantly negative in sample of firms with high pollution intensity, which is consistent with our reasoning. Columns 5–6 of Table 10 shows the results of the heterogeneity analysis by grouping firms by SO_2_ emissions. It shows that *EC* is not significant in the sample of firms with high pollution intensity. This suggests that the effectiveness of environmental courts differs based on the type of pollutants. As it is more challenging to monitor firm’ SO_2_ emissions and assign their legal liability compared to water pollution, environmental courts are found to be more effective in addressing water pollution. This explains why the negative impact of environmental courts on firm productivity is not significant in the sample of firms with high SO_2_ emissions, as the costs of environmental violations, environmental reputation and environmental compliance incurred by the environmental court are lower for these firms.

**Table 10:** Heterogeneity analysis (III).

	(1)	(2)	(3)	(4)	(5)	(6)
	HPI_IND	LPI_IND	HPI_WW	LPI_WW	HPI_SO_2_	LPI_SO_2_
	TFP	TFP	TFP	TFP	TFP	TFP
EC	-0.069***	-0.004	-0.067**	-0.010	-0.026	-0.048
	(-2.863)	(-0.127)	(-2.471)	(-0.272)	(-0.975)	(-1.523)
GDP	0.144	0.162	0.261**	0.035	0.202*	0.027
	(1.262)	(1.395)	(2.434)	(0.256)	(1.893)	(0.192)
FIA	0.049	-0.192	-0.078	-0.022	-0.017	-0.053
	(0.626)	(-1.585)	(-0.776)	(-0.213)	(-0.175)	(-0.469)
SED	-0.190	-0.134	-0.145	-0.330	-0.184	-0.120
	(-0.753)	(-0.438)	(-0.549)	(-1.122)	(-0.677)	(-0.395)
FDI	0.006	0.025	0.029*	0.018	0.011	0.009
	(0.292)	(1.183)	(1.728)	(1.017)	(0.799)	(0.442)
SIZE	0.358***	0.349***	0.306***	0.370***	0.322***	0.366***
	(13.806)	(12.510)	(11.513)	(12.745)	(12.174)	(12.963)
LEV	0.147	0.448***	0.177	0.328***	0.233**	0.260**
	(1.612)	(3.528)	(1.583)	(2.872)	(2.024)	(2.301)
TQ	0.053***	0.060***	0.067***	0.048***	0.055***	0.058***
	(5.055)	(5.203)	(5.570)	(5.211)	(4.649)	(5.918)
CF	0.233***	0.398***	0.436***	0.230**	0.369***	0.270***
	(2.626)	(3.839)	(4.574)	(2.544)	(3.902)	(3.014)
AGE	-0.097	0.026	-0.109	-0.159	-0.051	-0.197
	(-0.871)	(0.207)	(-0.902)	(-1.228)	(-0.406)	(-1.554)
BSIZE	0.093	-0.053	-0.022	-0.037	-0.043	-0.043
	(1.515)	(-0.722)	(-0.309)	(-0.349)	(-0.623)	(-0.400)
Constant	-0.612	-0.926	0.415	-0.801	0.320	-0.491
	(-0.919)	(-1.251)	(0.593)	(-1.129)	(0.466)	(-0.694)
FIRM	YES	YES	YES	YES	YES	YES
YEAR	YES	YES	YES	YES	YES	YES
N	4213	3870	3817	4042	3829	4044
Adj.R^2^	0.907	0.911	0.920	0.886	0.920	0.887
Test	0.066**		0.057**		-0.022	

Note: Table 10 reports the results of heterogeneity analysis. Robust t-statistics clustered at the firm level are reported in parentheses. ^*^, ^**^, and ^***^ denote significance at the 10%, 5%, and 1% levels, respectively. Test represents Fisher’s Permutation Test.

## 5. Further analysis

Our former study shows that environmental courts will reduce firm productivity in the short term. However, whether a harmonious equilibrium between environmental governance and economic performance could be achieved in the long term is a matter of concern. There could be an inter-temporal heterogeneity effect between environmental judicature and firm productivity. This is because, under the pressure of environmental judicature caused by environmental courts, firms may also overcome the short-sightedness of operational strategies and enhance green innovation to strengthen environmental governance. This improvement could lead to long-term benefits, as it takes time for firms to change strategies and achieve green transformation, eventually increase firm productivity. Thus, it is essential to examine the enduring impact of environmental courts on firm productivity. We try to examine how environmental courts affect firm productivity in the next one, two, three and four periods. Columns 1–4 of Table 11 show that environmental courts promote the next four periods of firm productivity.

**Table 11:** Further analysis.

	(1)	(2)	(3)	(4)	(5)	(6)
	TFP_t+1_	TFP_t+2_	TFP_t+3_	TFP_t+4_	GPAT_t+1_	GTFP_t+3_
EC	-0.038	0.012	0.050	0.143**	0.478*	0.047**
	(-1.374)	(0.310)	(1.122)	(2.340)	(1.702)	(2.046)
GDP	0.140	0.053	-0.131	-0.148	-0.125	0.004
	(1.476)	(0.528)	(-1.053)	(-1.246)	(-0.167)	(0.239)
FIA	0.058	0.139	0.142	-0.040	-0.280	0.008
	(0.692)	(1.201)	(1.142)	(-0.347)	(-0.521)	(0.507)
SED	-0.151	-0.374	-0.496*	-0.273	-1.279	0.000
	(-0.688)	(-1.442)	(-1.726)	(-0.971)	(-0.850)	(0.562)
FDI	-0.004	-0.027	-0.031	0.002	0.230**	-0.000
	(-0.169)	(-1.175)	(-1.235)	(0.113)	(2.404)	(-0.176)
HUMAN						0.003
						(0.452)
SIZE	0.384***	0.233***	0.104***	0.029	1.071***	
	(13.128)	(8.345)	(3.880)	(0.980)	(7.490)	
LEV	0.239***	0.231**	0.100	0.048	-0.914*	
	(2.709)	(2.560)	(1.116)	(0.515)	(-1.738)	
TQ	0.051***	0.042***	0.037***	0.044***	0.050	
	(6.101)	(3.887)	(3.565)	(4.057)	(0.857)	
CF	0.341***	0.014	0.068	0.105	0.930*	
	(4.351)	(0.166)	(0.821)	(1.197)	(1.709)	
AGE	-0.151	0.044	0.113	0.030	0.983	
	(-1.202)	(0.302)	(0.692)	(0.161)	(1.216)	
BSIZE	-0.037	-0.083	0.034	0.043	-0.314	
	(-0.614)	(-1.301)	(0.500)	(0.603)	(-0.841)	
Constant	-0.621	2.964***	5.606***	6.778***	-26.505***	1.018***
	(-0.962)	(4.156)	(7.043)	(8.144)	(-6.619)	(13.015)
FIRM	YES	YES	YES	YES	YES	NO
YEAR	YES	YES	YES	YES	YES	YES
CITY	NO	NO	NO	NO	NO	YES
N	5941	4627	3804	3113	5941	2079
Adj.R^2^	0.910	0.903	0.904	0.907	0.561	0.106

Note: Table 11 reports the results of further analysis. Columns 1–5 show the results of the firm panel data regression, and Column 6 shows the the result of the city panel data regression. Robust t-statistics clustered at the firm level or city level are reported in parentheses. ^*^, ^**^, and ^***^ denote significance at the 10%, 5%, and 1% levels, respectively.

We further test whether the beneficial impacts of environmental courts are mediated through the green innovation channel. Following the previous literature [52], we employ the natural logarithm of one plus the number of green patent applications in a year to measure firm green innovation (*GPAT*). It shows that environmental courts do promote the next one period of firm green innovation in Columns 5 of Table 11, indicating that environmental courts are conducive to combating the short-sightedness of operational strategies, force firms to strengthen environmental governance by enhancing green innovation and accelerating green transformation.

If environmental courts are conducive to firm innovation in the long term, indicating that they are the main driver of firm green transformation, we argue that environmental courts should be helpful in improving the future green productivity at city level. Following the previous literature [53], we calculate green productivity (*GTFP*) at city level, and investigate the effect of environmental courts on green productivity in the next three period. We additionally control for cities’ human capital (*HUMAN*) in the regression. *HUMAN* is measured by the natural logarithm of the number of students in ordinary colleges and universities in city *c* in year *t*. Column 6 of Table 11 reports the results. It shows that environmental courts promote the next three period of green productivity at city-level, indicating the environmental courts are conducive to the green transformation of the region in the long term.

## 6. Conclusions and policy implications

Using the establishment of environmental courts as a quasi-natural experiment, this paper examines the relationship between environmental judicature and firm productivity. We find that: (1) Environmental courts will reduce firm productivity in the short term, chiefly owing to the increased environmental violation costs, environmental reputation costs and environmental compliance costs. The conclusion remains robust after mitigation of heterogeneous treatment effects, PSM-DID estimation, placebo tests and IV estimation. (2) This negative effect is more pronounced for firms located in regions with high legal scores and low government intervention, for firms located in the eastern region, for firms with weak market power, and for firms with high pollution intensity. (3) Our further analysis finds that the environmental courts would improve the long-term firm productivity and regional green productivity, indicating that the environmental courts are conducive to firms’ enhancement of productivity and regions’ green transformation in the long term. The policy implications are as follows:

For legal institutions, despite the substantial achievements brought about by the environmental courts, some successful experiences need to be further built upon. Firstly, considering the huge role played by public supervision in environmental protection, there is also a need to further improve environmental legislation to safeguard the legal status of the public in environmental supervision. Secondly, it is essential to further deepen the reform of environmental judicature. Through the specialization of environmental trial mechanism, procedure, trial theory and trial team, the environmental judicial efficiency could be further improved.

For local governments, there is a need to reduce government interference in the judiciary while creating a favourable business environment. On the one hand, reducing government intervention in the judiciary is conducive to maintaining a level playing field in the market. Enterprises that are sheltered by the government will be forced to implement a green transformation to achieve environmental compliance and also compete on a level playing field with non-sheltered enterprises, thus improving firm productivity in the long term. On the other hand, the local government should introduce some financial support policies, such as establishing a financial subsidy system and perfecting the policy of tax preferences. Appropriate financial support policies will offset firms’ environmental cost burden, which is helpful in mitigating the negative effect of environmental courts.

For firms, it is crucial to strengthen technological innovation, process innovation and product innovation to gain competitive advantage in order to mitigate cost shocks triggered by environmental judicature. In addition, firms not only need environmental judicature as an external governance mechanism, but also need to strengthen their internal corporate governance mechanisms to mitigate management short-sightedness, enhance firm innovation and promote green transformation.
